# Identification of tumorigenicity-associated genes in osteosarcoma cell lines based on bioinformatic analysis and experimental validation

**DOI:** 10.7150/jca.37393

**Published:** 2020-03-26

**Authors:** Shaojie Jiang, Fei Zhou, Yanhua Zhang, Weiping Zhou, Linghua Zhu, Miaofeng Zhang, Jingfeng Luo, Rui Ma, Xiufang Xu, Jiying Zhu, Xue Dong, Shuangling Zhang, Jie Fang, Jihong Sun, Xiaoming Yang

**Affiliations:** 1 Department of Radiology, Sir Run Run Shaw Hospital, School of Medicine, Zhejiang University, Hangzhou, Zhejiang, 310016, China.; 2 School of Medical Imaging, Hangzhou Medical College, Hangzhou, Zhejiang, 310013, China.; 3 Department of Pathology, Sir Run Run Shaw Hospital, School of Medicine, Zhejiang University, Hangzhou, Zhejiang, 310016, China.; 4 Department of Diagnostic Ultrasound and Echocardiography, Sir Run Run Shaw Hospital, School of Medicine, Zhejiang University, Hangzhou, Zhejiang, 310016, China.; 5 Department of General Surgery, Sir Run Run Shaw Hospital, School of Medicine, Zhejiang University, Hangzhou, Zhejiang, 310016, China.; 6 Department of Orthopedic Surgery, Second Affiliated Hospital, School of Medicine, Zhejiang University, Hangzhou, Zhejiang, 310009, China.; 7 Department of Surgery, Zhejiang University Hospital, Zhejiang University, Hangzhou, Zhejiang 310027, China; 8 Key Laboratory of Experimental Animal and Safety Research, Zhejiang Academy of Medical Sciences, Hangzhou, Zhejiang 310013, China.; 9 Image-Guided Bio-Molecular Intervention Research, Department of Radiology, University of Washington School of Medicine, Seattle, Washington, 98109, USA.

**Keywords:** osteosarcoma, bioinformatic analysis, tumorigenicity, HEY1, prognosis

## Abstract

Osteosarcoma is the most common primary malignant tumor of bone. Tumorigenic investigation of osteosarcoma cell lines may facilitate preclinical studies of targeted therapy. Therefore, the aim of this study was to explore the tumorigenicity-associated genes in osteosarcoma cells. We found that 138 genes were highly expressed and 86 genes were lowly expressed in highly tumorigenic osteosarcoma cell lines (143B, MNNG/HOS, and SJSA-1) compared with poorly tumorigenic osteosarcoma cell lines (MG-63, Saos-2, and U-2 OS). Kyoto Encyclopedia of Genes and Genomes (KEGG) analysis revealed that highly expressed genes were associated with amino acids and energy metabolism, while lowly expressed genes were associated with cell cycle and DNA replication. Gene Ontology (GO) analysis showed that highly expressed genes were associated with endoplasmic reticulum stress response and aggrephagy, whereas lowly expressed genes were correlated with extracellular matrix assembly and DNA damage response. Further analysis identified six highly expressed genes and six lowly expressed genes. Three of highly expressed genes (*DDX10*, *FOXA2*, and *HEY1*) were correlated with poor prognosis, while three of lowly expressed genes (*CYP26B1*, *GP1BB*, and *IFI44*) showed the opposite trend in patients with osteosarcoma. Knockdown of *HEY1* significantly inhibited the tumorigenicity of 143B cells in BALB/c nude mice.

## Introduction

Osteosarcoma is the most common primary malignant tumor of bone that most commonly affects children, adolescents, and young adults, with a global incidence of approximately one to three cases per million annually 1, 2. Surgical resection was the primary treatment before 1970. However, the survival of osteosarcoma patients treated with surgery alone is less than 20% 3. The long-term survival rate was improved to 65-70% after the advent of multiagent chemotherapy regimens in the early 1970s, especially the combination of high-dose methotrexate, doxorubicin, and cisplatin (MAP) 4.

Osteosarcoma exhibits a predilection for the long bones near the metaphyseal growth plate and most commonly occurs in the distal femur (43%), proximal tibia (23%), or humerus (10%) 1, 2, 5. The incidence is higher in adolescents of age 15-19, with eight to eleven cases per million annually, accounting for >10% of all solid cancers 6. The patients with localized osteosarcoma that respond to preoperative combination chemotherapy always show good prognosis 7. However, the overall 5‑year survival rate for patients with metastatic or relapsed osteosarcoma has remained at about 20% over the past 30 years 8. The lung is the most common site of osteosarcoma metastasis (more than 85% of cases), with bone the second most common site 5. Thus, seeking specific targets for early diagnosis and effective treatment is necessary.

Although candidate oncogenes (e.g., *RUNX2*, *VEGFA*, *MDM2*, and *PRIM1*) 9-12 and tumor suppressors (e.g., *TP53*, *RB1*, and *PTEN*) 13-15 have been identified in human osteosarcoma, the search for common molecular therapeutic targets in osteosarcoma has been disappointing to date 2. Current clinical trials are focused on targeting the bone microenvironment (mainly osteoclasts and bone seeking) 8, 16, receptor tyrosine kinases (including Kit, VEGFR, FGFR, PDGFR, HER2, and IGF1R) 17-19, intracellular signaling pathways (SRC, mTOR, RAF, Aurora kinase A, and histone deacetylase) 20, and immune system 21-23. Mifamurtide as an innate immune stimulant has shown clinical promise in the treatment of osteosarcoma 22, 23. However, the strategies that target the bone microenvironment and signaling pathways remain controversial. For example, the Hedgehog, Notch, and WNT pathways have been implicated in both osteosarcoma and normal bone development, and targeted therapy may be problematic for children.

Tumorigenicity is defined as the ability of cells to give rise to tumors. Tumorigenicity of osteosarcoma cell lines in nude mouse is an important method for candidate target and drug screening. However, the mechanism remains obscure. Herein, the tumorigenic genes in osteosarcoma cell lines were identified by bioinformatic analysis, and Hes related family bHLH transcription factor with YRPW motif 1 (*HEY1*) was identified as a key factor in tumorigenicity of osteosarcoma cells by subsequent experimental validation.

## Methods

### Cell lines

Six osteosarcoma cell lines were obtained from American Type Culture Collection (ATCC) (https://www.atcc.org/). SJSA-1 (also known as OSA, ATCC: CRL-2098) and U-2 OS (ATCC: HTB-96) were cultured in RPMI-1640 (Gibco, 11875-085) containing 10% fetal bovine serum (FBS) (Gibco, 1099-141); 143B (ATCC: CRL-8303), MlNNG/HOS Cl #5 [R-1059-D] (ATCC: CRL-1547), and MG-63 (ATCC: CRL-1427) were cultured in minimum essential medium (MEM) (Gibco, 11095-080) containing 10% FBS; Saos-2 (ATCC: HTB-85) was cultured in McCoy's 5A (Gibco, 16600-082) containing 15% FBS. All cell lines were maintained at 37℃ with 5% CO_2_.

### *In vivo* tumorigenicity

Osteosarcoma cell lines MG-63, Saos-2, U-2 OS, 143B, MNNG/HOS, and SJSA-1 were used for xenograft transplantation in BALB/c nude mice according to a previous report 24. Briefly, 1×10^6^ cells in 100 μL serum-free phosphate-buffered saline (PBS) were injected subcutaneously into each flank of locally bred BALB/c-nu mice (5 weeks old, female; five mice per group). The tumor size was measured every 5 days, and tumor volume was calculated by the formula (length×width×width)/2. For the highly tumorigenic cell lines, the experiments were stopped within a month. For the poorly tumorigenic cell lines, the experiments were stopped after 90 days.

### Quantitative polymerase chain reaction (qPCR) analysis

Total cellular RNA was extracted from osteosarcoma cells or stable shRNA-expressing 143B cells (80-90% confluence) using TRIzol reagent (Invitrogen, 15596026). The RNA concentration was measured by the NanoDrop 2000 (Thermo Fisher Scientific, ND2000). The first-strand cDNA was synthesized from 2 μg of total RNA with random primers, and subsequent qPCR was performed using the GoTaq RT-qPCR kit (Promega, A6010). The experiment was carried out with the ViiA 7 Real-Time PCR System (Applied Biosystems, 4453534), and expression of mRNA was assessed based on the threshold cycle (Ct). The relative mRNA expression levels were calculated as 2-^[(Ct of mRNA)-(Ct of *GAPDH*)]^ after normalization to *GAPDH* expression. qPCR primer sequences (F, forward; R, reverse; 5′→3′) were as follows: *CYP26B1*-F: GTTCTGCCTCGGAGCTGATT, *CYP26B1*-R: CAACCCAATCCCCCTGACAA; *GP1BB* -F: AGACCACGTGGGACAGAACT, *GP1BB*-R: CAGGGTCTGGACCGCATTG; *IFI44*-F: TGGGAGCTGGACCCTGTAAA, *IFI44*-R: CCTCCCTTAGATTCCCTATTTGCT; *DDX10*-F: ATGTACTTGGAGCGGCCAAA, *DDX10*-R: CCCAGCCCATCTGTTGAAGT; *FOXA2*-F: CTTCAAGTGCGAGAAGCAGC, *FOXA2*-R: CCGAGTTGAGCCTGTGAGG; *HEY1*-F: AGTTAGGAGAGAGCCGCTGA, *HEY1*-R: TGTTGCTGGGGCTGGTAAAT; *GAPDH*-F: AGGTCGGAGTCAACGGATTT; *GAPDH*-R: ATGAAGGGGTCATTGATGGCA.

### Virus production and transfection

shRNAs targeting *HEY1* were cloned into the pLent-U6-RFP-Puro (Vigne, #LT88024) vector. Targeting sequences were as follows: shRNA-Ctr: 5′-GCACCCAGUCCGCCCUGAGCAAA-3′; shHEY1-1: 5′-GCAGGAGGGAAAGGUUACUUU-3′; shHEY1-2: 5′-CCCAACUACAUCUUCCCAGAU-3′. The 293T cell line was used for lentivirus packaging. Briefly, a 10-cm dish of 4×10^6^ non-confluent 293T cells were co-transfected with recombinant pLent-U6-RFP-Puro (Vigene, LT88024), pMD2G (Addgene, #12259), and psPAX2 (Addgene, #12260) for knock-down (RNAi), and pLent-C-HA (Vigene, LT88007), pMD2G, and psPAX2 for overexpression. The lentivirus-containing supernatant was harvested after 48 h and used for the subsequent experiment.

### Western blot analysis

All cell lines were harvested at 80-90% confluence and then lysed in radioimmunoprecipitation assay (RIPA) buffer (Thermo Fisher Scientific, #89901). Protein concentration was measured with a bicinchoninic acid (BCA) protein assay kit (Thermo Fisher Scientific, #23225). Protein samples were resolved by 8-10% sodium dodecyl sulfate-polyacrylamide gel electrophoresis (SDS-PAGE) and transferred to a polyvinylidene difluoride (PVDF) membrane (Bio-Rad, #162-0117). The membrane was blocked in Tris-buffered saline (TBS) containing 0.05% Tween-20 (Amresco, 0777-1L) with 5% nonfat skim milk (BD, #232100) for 1 h at room temperature, followed by overnight incubation with primary antibodies at 4°C. After three washes in TBS with Tween-20 (TBST), the membrane was incubated with horseradish peroxidase (HRP)-conjugated secondary antibody for 1 h at room temperature. After three washes in TBST, the membrane was treated with the EZ-ECL kit (Biological Industries, #20-500-120) and visualized using the Tanon-5200 multi-automatic chemiluminescence/fluorescence imaging analysis system (Tanon Science & Technology Inc). The following antibodies were used: anti-HEY1 (Abcam, ab154077), anti-β-actin (CST, #3700), and secondary antibodies including anti-rabbit IgG, HRP-linked antibody (CST, #7074) and anti-mouse IgG, HRP-linked antibody (CST, #7076).

### *In vivo* fluorescence imaging

Images of 143B cell-bearing mice were acquired using the Clairvivo OPT plus imaging system (SHIMADZU). Briefly, mice were anesthetized by 2% isoflurane gas for 5 min using the RC2 Anesthesia Machine (VetEquip). Then, postanesthetic mice were imaged under 530-nm laser irradiation at the experimental endpoint.

### Microarray datasets

The microarray datasets in this study were obtained from the Gene Expression Omnibus (GEO, http://www.ncbi.nlm.nih.gov/geo/). Gene expression profiles (GSE36001 and GSE42352) were provided by Professor Leonardo A. Meza-Zepeda 25 and Professor Anne-Marie Cleton-Jansen 26, respectively. Both GSE36001 and GSE42352 contain the gene expression profiles of osteosarcoma cell lines MG-63, Saos-2, U-2 OS, 143B, MNNG/HOS, and SJSA-1 (or OSA). The Sanger software package (V1.0.8) (https://shengxin.ren/softs/Sanger_V1.0.8.zip) was used for volcano plot calculation and visualization.

### Signaling pathway, biological process, and protein-protein interaction (PPI) network enrichment analyses

For signaling pathway and biological process enrichment analyses, the high-expression group containing 138 genes and low-expression group containing 86 genes in highly tumorigenic osteosarcoma cell lines (143B, MNNG/HOS, and SJSA-1) as compared with poorly tumorigenic osteosarcoma cell lines (MG-63, Saos-2, and U-2 OS) were analyzed using a publicly accessible web-based platform: Enrichr (http://amp.pharm.mssm.edu/Enrichr/) 27, 28. Signaling pathway enrichment analysis was performed by Kyoto Encyclopedia of Genes and Genomes (KEGG) pathway analysis, while biological process enrichment analysis was performed by gene ontology (GO) analysis. For PPI network enrichment and gene function analyses, STRING (https://string-db.org/) was used.

### Kaplan-Meier analysis

Kaplan-Meier survival plots were generated using the R2: Genomics Analysis and Visualization Platform (http://r2.amc.nl). Briefly, osteosarcoma patients were divided into two groups based on the expression value of indicated genes, and the survival rate between the two groups was tested via log-rank test with P-value<0.05 considered significant.

### Statistical analysis

All data were analyzed with GraphPad Prism 6 software and are provided as the mean ± standard error of the mean (SEM) unless otherwise indicated. Statistical analyses were performed using an unpaired Student's t-test. P<0.05 was considered to indicate statistical significance.

## Results

### Tumorigenicity assay of human osteosarcoma cell lines

**Figure 1:**
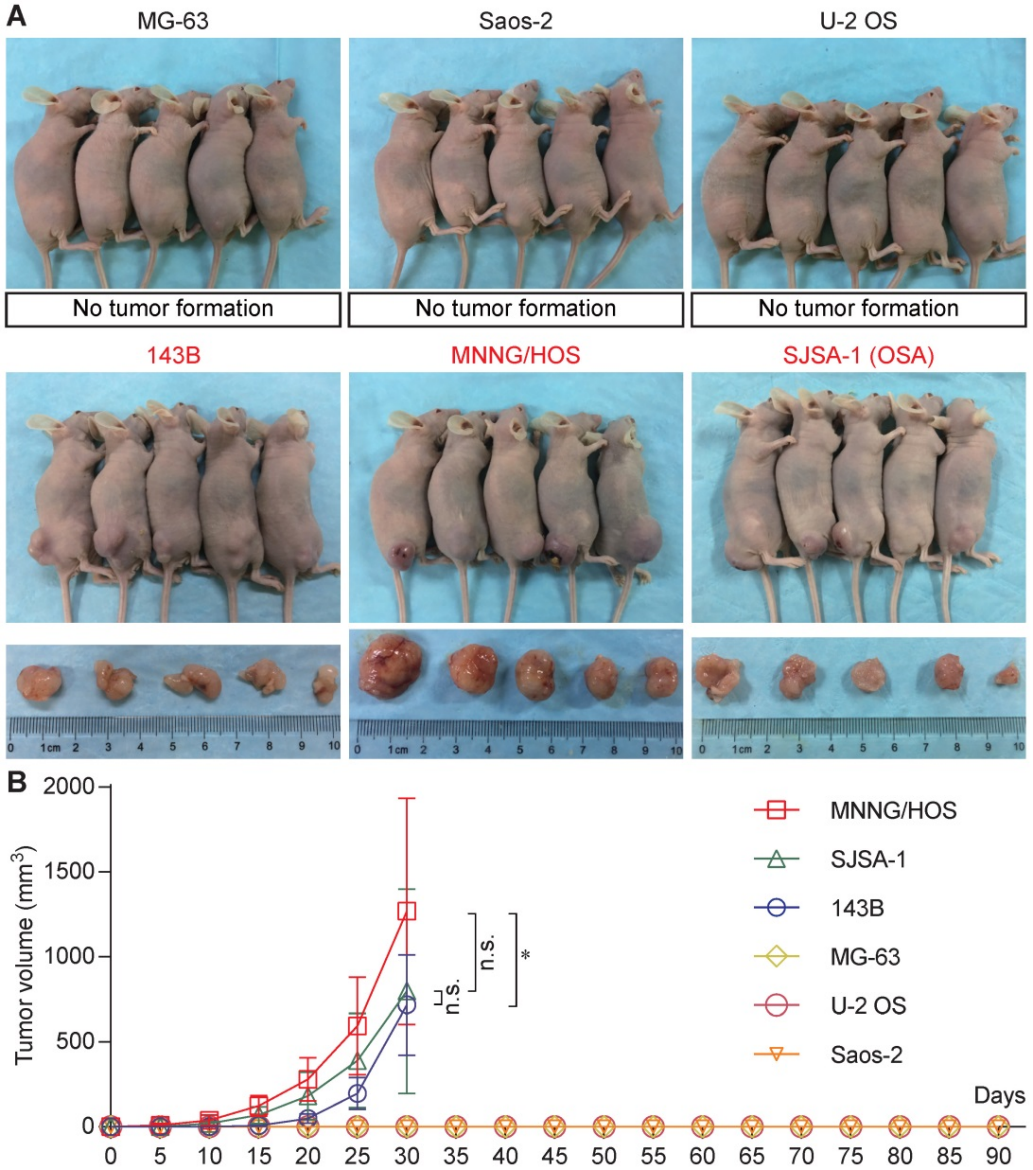
Tumorigenicity assay of human osteosarcoma cell lines. **(A)** Tumorigenicity of six osteosarcoma cell lines (MG-63, Saos-2, U-2 OS, 143B, MNNG/HOS, and SJSA-1) *in vivo*. **(B)** Tumor growth curve based on tumor volume (*P<0.05, n.s.: no significant by two-way ANOVA).

Six osteosarcoma cell lines (MG-63, Saos-2, U-2 OS, 143B, MNNG/HOS, and SJSA-1) were used for tumorigenicity testing in BALB/c nude mice. At 30 days, all five mice formed tumors with 143B, MNNG/HOS, and SJSA-1cells, while none of the five mice formed tumors with MG-63, Saos-2, or U-2 OS cells (Figure 1A). The 143B, MNNG/HOS, and SJSA-1 cell lines formed tumors very rapidly, reaching a volume of ~1,000 mm^3^ within 30 days. In addition, MNNG/HOS showed the highest tumorigenicity compared with 143B and SJSA-1 (Figure 1B).

### GEO and PPI analyses

**Figure 2:**
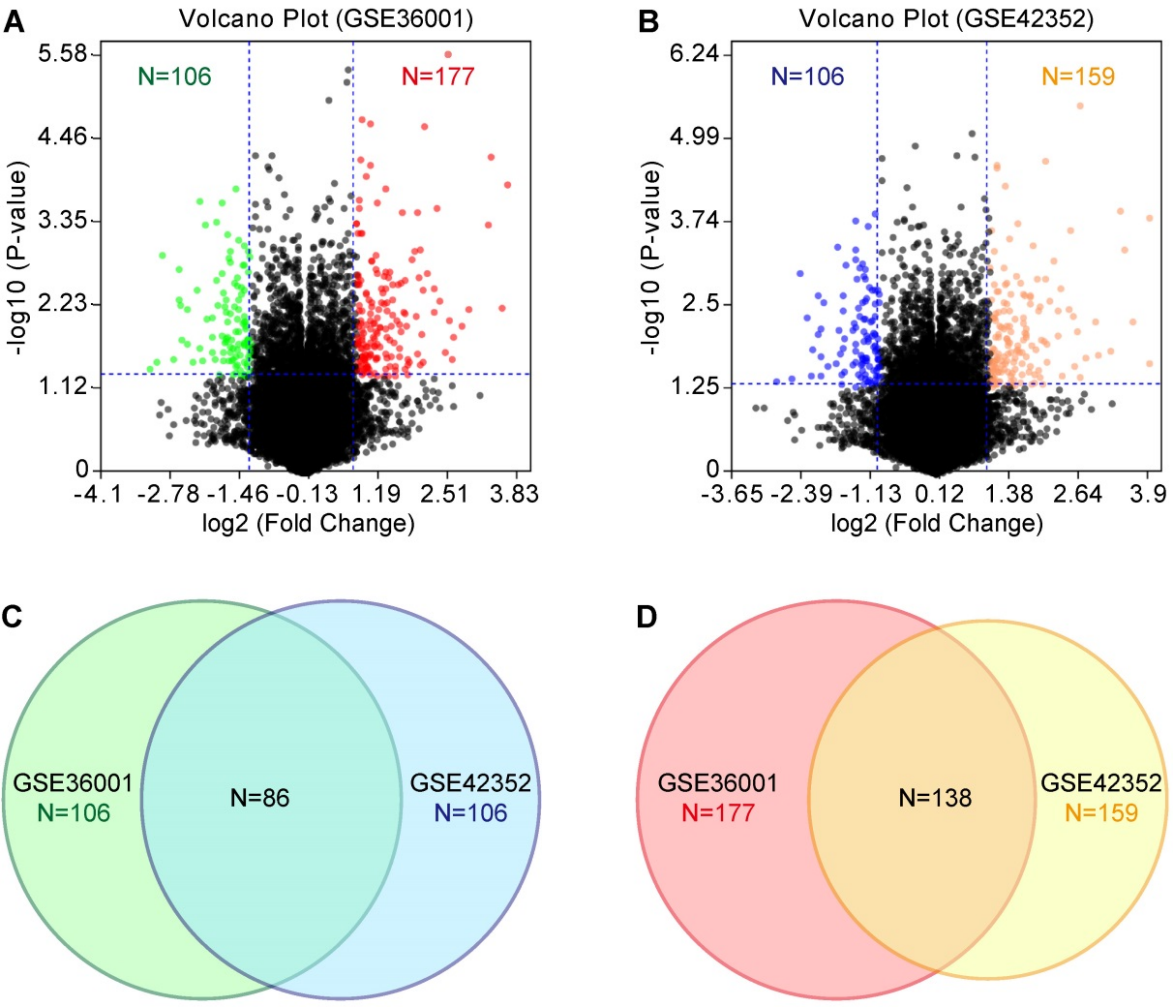
GEO analysis of differentially expressed genes. **(A)** & **(B)** Volcano plot of differentially expressed genes based on GSE36001 and GSE42352, respectively. **(C)** Differentially lowly expressed genes. **(D)** Differentially highly expressed genes.

According to the tumorigenicity assay, the six osteosarcoma cell lines were divided into the highly tumorigenic group (143B, MNNG/HOS, and SJSA-1) and lowly tumorigenic group (MG-63, Saos-2, and U-2 OS). GEO datasets (GSE36001 and GSE42352) were analyzed with the GEO2R online tool. We set the threshold as P-value<0.05 and log2 fold change (FC)>1. Compared with the lowly tumorigenic group, 106 and 106 lowly expressed genes and 177 and 159 highly expressed genes were identified from GSE36001 and GSE42352, respectively, in the highly tumorigenic group (Figure 2A & B, Table S1). By combining GSE36001 and GSE42352, 86 lowly expressed genes and 138 highly expressed genes were identified (Figure 2C & D, Table S1). PPI analysis showed that 53 of 86 lowly expressed genes (Figure S1) and 89 of 137 highly expressed genes (Figure S2) were not included in the main PPI network, although the PPI enrichment P-value = 1.67e-05 for lowly expressed genes and 0.00961 for highly expressed genes. The low physical protein-protein interaction of these genes suggested that tumorigenicity involves various biological processes.

### Signaling pathway (KEGG) and biological process (GO) enrichment analyses

**Figure 3:**
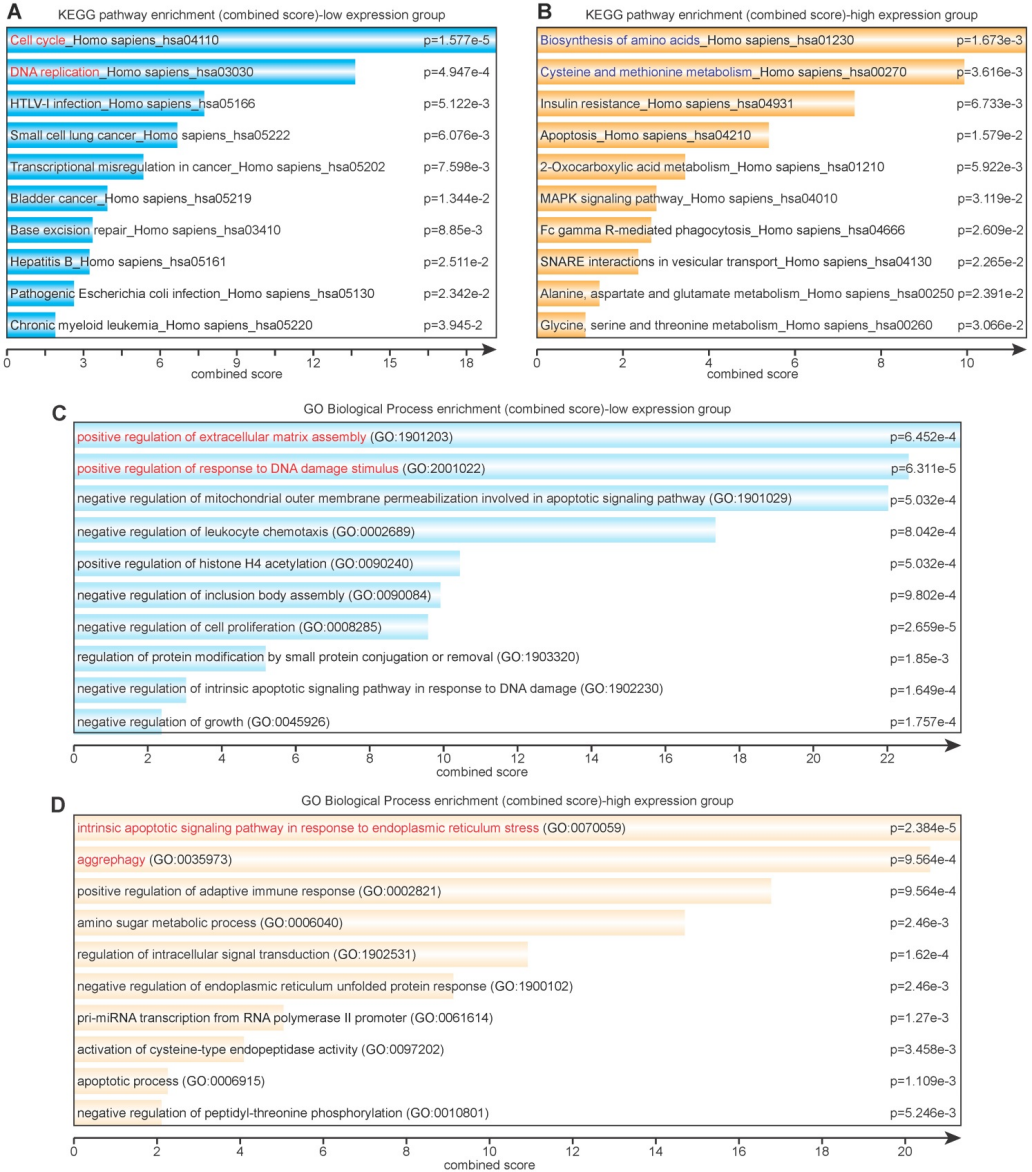
Signaling pathway (KEGG) and biological process (GO) enrichment analyses. **(A)** KEGG analysis of differentially lowly expressed genes. **(B)** KEGG analysis of differentially highly expressed genes. **(C)** GO analysis of differentially lowly expressed genes. **(D)** GO analysis of differentially highly expressed genes.

Eighty-six lowly expressed genes and 138 highly expressed genes were subjected to further KEGG and GO pathway analysis via Enrichr. The KEGG enrichment analysis revealed that lowly expressed genes were largely correlated with cell cycle, DNA replication and repair, virus infection, and various cancers (Figure 3A), whereas highly expressed genes were correlated with amino acid metabolism and glucose metabolism (Figure 3B). The GO enrichment analysis showed that lowly expressed genes corresponded with positive regulation of extracellular matrix assembly and response to DNA damage stimulus and negative regulation of mitochondrial outer membrane permeabilization, cell proliferation, and growth (Figure 3C); highly expressed genes corresponded with response to endoplasmic reticulum (ER) stress, aggrephagy, positive regulation of adaptive immune response, and amino sugar metabolic process (Figure 3D).

### Identification of candidate lowly expressed and highly expressed genes

**Figure 4:**
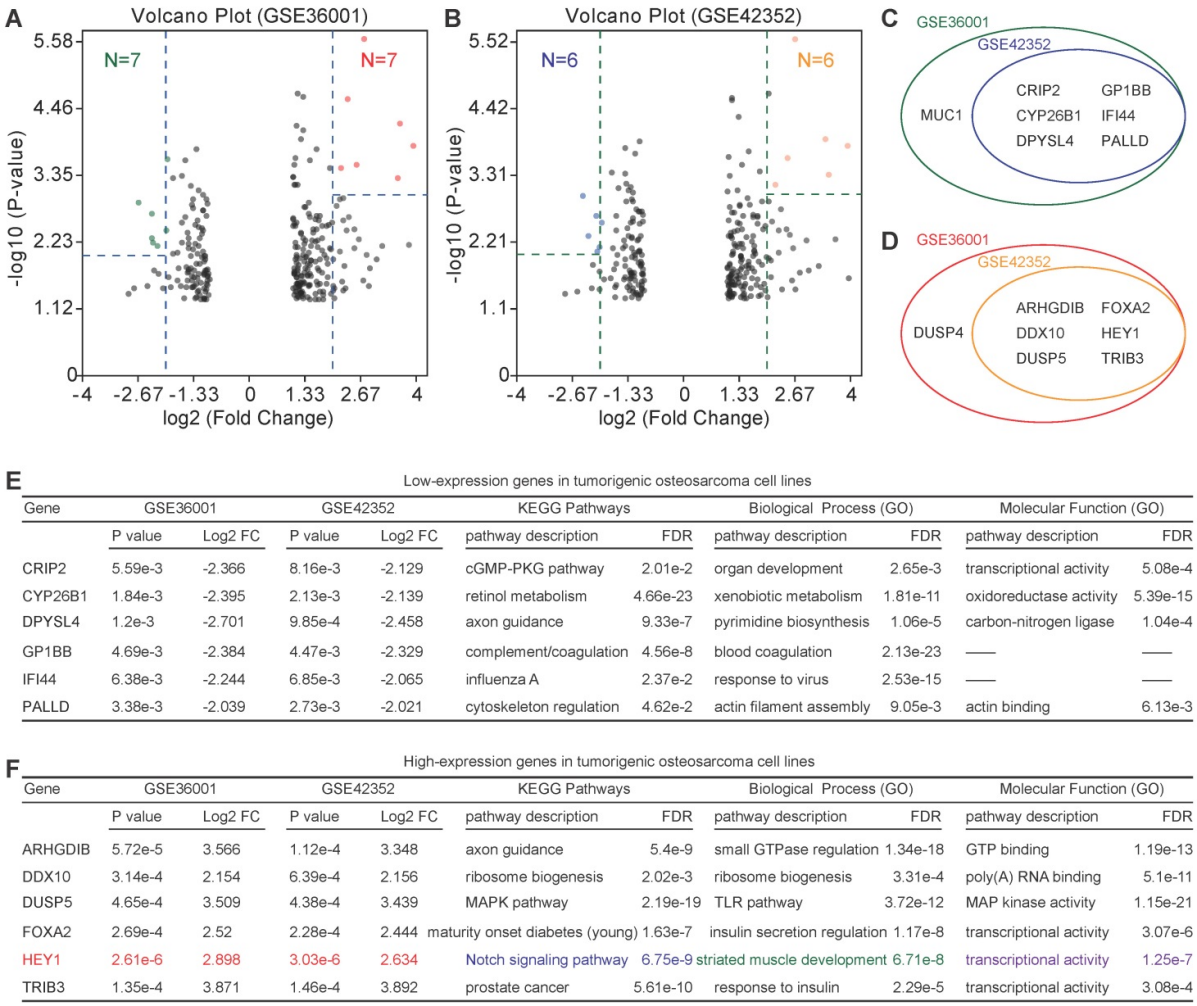
Identification of candidate lowly expressed and highly expressed genes. **(A)** & **(B)** Volcano plot of candidate lowly expressed and highly expressed genes. **(C)** & **(D)** Lowly and highly expressed genes. **e** Function enrichment of lowly expressed genes. **f** Function enrichment of highly expressed genes. Log2 FC: Log2 fold change; FDR: False discovery rate.

Because of the low relevance of differentially expressed genes, further enrichment analysis was performed. For highly expressed genes, we set the threshold as P-value<0.01 and log2FC>2, and for lowly expressed genes, we set the threshold as P-value<0.005 and log2FC>2. Six lowly expressed genes (*CRIP2*, *CYP26B1*, *DPYSL4*, *GP1BB*, *IFI44*, and *PALLD*) and 6 highly expressed genes (*ARHGDIB*, *DDX10*, *DUSP5*, *FOXA2*, *HEY1*, and *TRIB3*) were identified (Figure 4A-D). However, the functions of these genes were quite different according to the PPI analysis (PPI enrichment P-value = 1) (Figure 4E & F). Of note, *HEY1* had an extremely low P-value in both GSE36001 and GSE42352.

### Prognostic analysis and qPCR validation

**Figure 5:**
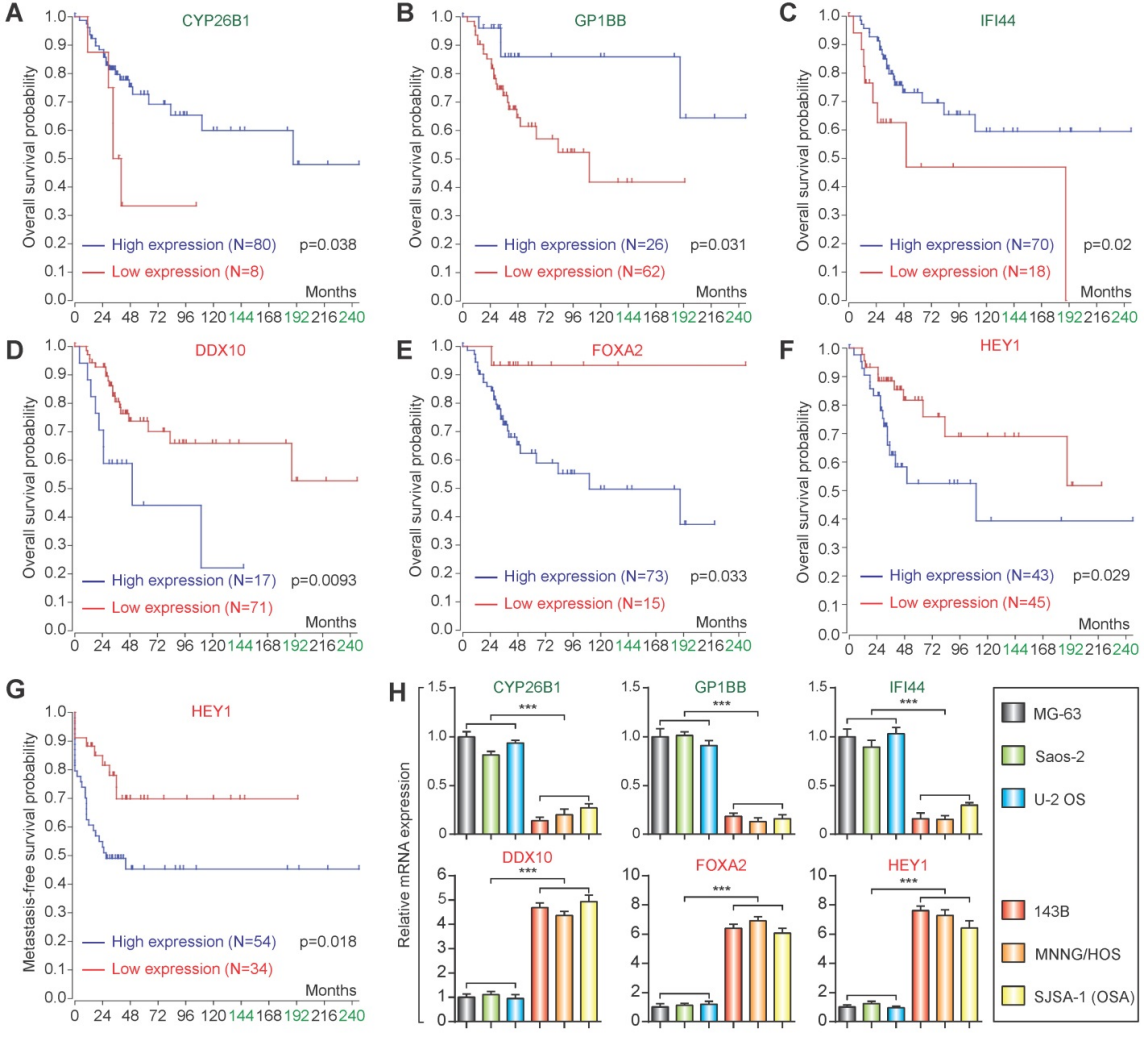
Prognostic analysis and qPCR validation. Overall survival analysis of *CYP26B1* **(A)**, *GP1BB* **(B)**, *IFI44* **(C)**, *DDX10* **(D)**, *FOXA2* **(E)** and *HEY1* **(F)**. **(G)** Metastasis-free survival of* HEY1*. **(H)** qPCR validation of identification of candidate lowly expressed and highly expressed genes. (***P<0.001 by student's t-test).

Based on the candidate lowly expressed and highly expressed genes, the R2 Genomics Platform was used for prognostic analysis. High expression of *CYP26B1*, *GP1BB*, and *IFI44* in osteosarcoma patients was correlated with good prognosis (Figure 5A-C), while high expression of *DDX10* and *FOXA2* in osteosarcoma patients was associated with poor prognosis (Figure 5D & E). Notably, high expression of *HEY1* corresponded with poor prognosis (both overall survival and metastasis-free survival) (Figure 5F & G). qPCR confirmed that the expression changes were consistent with the microarray datasets (Figure 5H).

### Importance of HEY1 in the tumorigenicity of 143B osteosarcoma cells

**Figure 6:**
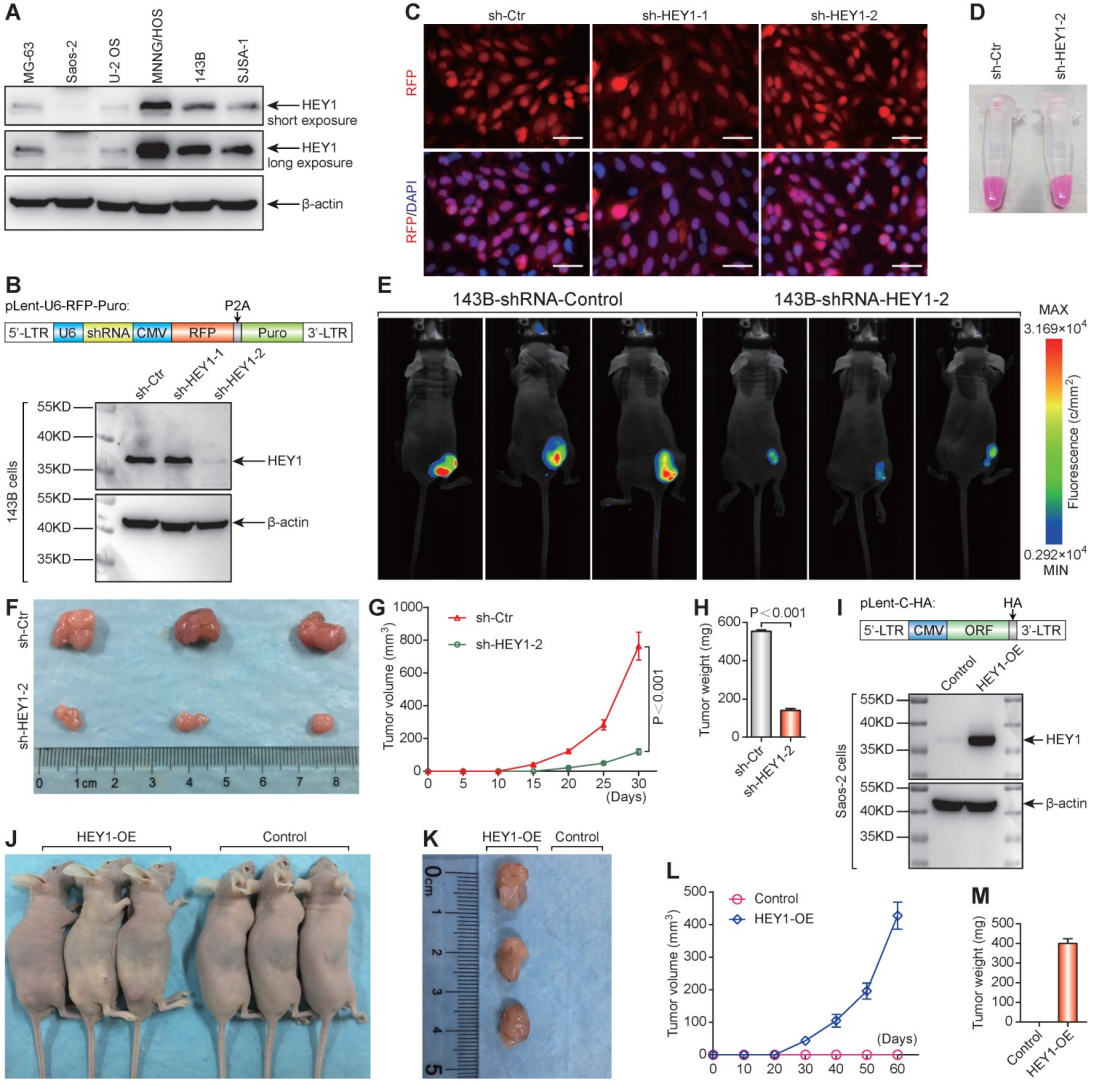
HEY1 is a key factor in the tumorigenicity of 143B osteosarcoma cells. **(A)** Protein expression level of HEY1 in six osteosarcoma cell lines (MG-63, Saos-2, U-2 OS, MNNG/HOS, 143B, and SJSA-1). **(B)** Protein expression level of HEY1 after lentivirus-mediated RNA interference in 143B osteosarcoma cells. **(C)** Lentivirus infection efficiency detected by fluorescence microscopy (scale bar = 200 μm). **(D)** Lentivirus-infected 143B osteosarcoma cell pellet under visible light. **(E)** Images of 143B cell-bearing mice under 530-nm laser irradiation at the experimental endpoint. Subcutaneous tumors at the experimental endpoint **(F)**, tumor growth curves **(G)**, and tumor weight **(H)** are shown. **(I)** Protein expression level of HEY1 in Saos-2 and HEY1-overexpressed Saos-2 cells. **(J)** Tumorigenicity assay of Saos-2 cells after HEY1-overexpression. Subcutaneous tumors at the experimental endpoint **(K)**, tumor growth curves **(L)**, and tumor weight **(M)** of Saos-2 cells after HEY1-overexpression are shown.

The protein expression level of HEY1 detected by western blot was consistent with the mRNA level in osteosarcoma cell lines (Figure 6A). Western blot analysis of changes in HEY1 expression showed that shHEY1-2 is more effective (Figure 6B) under an equivalent infection rate (Figure 6C). The 143B osteosarcoma cells with red fluorescent protein (mStrawberry) were visualized under visible light (Figure 6D) to monitor cell growth (Figure 6E). The appearance of subcutaneous tumors at the experimental endpoint (Figure 6E & F), tumor growth curves (Figure 6G), and tumor weight (Figure 6H) showed that knockdown of HEY1 mRNA inhibited the tumorigenicity significantly. On the other hand, overexpression of HEY1 in a non-tumorigenic cell line Saos-2 (Figure 6I) could help Saos-2 cells to obtain the tumorigenicity (Figure 6J-M). These results revealed that *HEY1* is a key factor in the tumorigenicity of 143B osteosarcoma cells.

## Discussion

Identifying the tumorigenicity-associated genes in osteosarcoma is a valuable method for investigation of tumorigenic mechanisms. In this study, we evaluated the tumor-forming ability of six osteosarcoma cell lines. Three (143B, MNNG/HOS, and SJSA-1) were found to be tumorigenic, while the other three cell lines (MG-63, Saos-2, and U-2 OS) lacked tumorigenicity (Figure 1A). Thus, 143B, MNNG/HOS, and SJSA-1 were defined as the highly tumorigenic group, while MG-63, Saos-2, and U-2 OS were defined as the poorly tumorigenic group. Two microarray datasets from two independent studies containing the mRNA expression data of these six cell lines were used for analysis of differentially expressed genes.

It is generally accepted that the genes that promote cell cycle progression and DNA replication act as positive factors in tumorigenesis 29. However, tumorigenicity is not always correlated with tumorigenesis or proliferation. The poorly tumorigenic MG-63 and U-2 OS cells showed the same proliferation rate as the highly tumorigenic 143B and SJSA-1 cells 24. KEGG analysis showed that the cell cycle- and DNA replication-related genes were lowly expressed in the highly tumorigenic group, while amino acid metabolism- and glucose metabolism- related genes were highly expressed in the highly tumorigenic group (Figure 3A & B). Thus, we speculate that amino acid- and energy metabolism- associated genes are important to the tumorigenicity. In contrast, vigorous metabolism may help the osteosarcoma cells adapt to the environmental alteration 30. Coincidentally, GO analysis showed that the intrinsic apoptosis- and aggrephagy-related genes were highly expressed in the highly tumorigenic group. It may help osteosarcoma cells overcome the temporary nutrition deficiency and adapt to the bioenergetic challenges *in vivo* 31, 32.

PPI analysis showed low relevance among the differentially expressed genes, which suggests that tumorigenicity is a complicated process involving various genes and biological pathways. Additional enrichment analysis identified six lowly expressed and six highly expressed genes. Nonetheless, there was no relevance among these differentially expressed genes. Half of these differentially expressed genes were correlated with good or poor prognosis. Indeed, one of the shortcomings of prognosis analysis for rare pediatric tumors, such as this study, is the lack of adequate samples to power the discovery of rare events.

We noticed that *HEY1* was highly expressed in the highly tumorigenic group with an extremely low P-value in both GSE36001 and GSE42352. In addition, the expression level of *HEY1* was positively correlated with the tumorigenicity of osteosarcoma cell lines. Thus, *HEY1* was selected for further investigation. In fact, *HEY1* is a candidate oncogene that is highly expressed in glioblastoma 33, 34, rhabdomyosarcoma 35, hepatocellular carcinoma 36, head and neck squamous cell carcinoma 37, and renal cell carcinoma 38, among other cancers. It is also associated with poor prognosis in head and neck squamous cell carcinoma 37 and esophageal squamous cell carcinoma 39. On the other hand, *HEY1* acts a target gene of Delta-like canonical Notch ligand 4 (Dll4)-induced Notch signaling pathway activation and functions as an inhibitor of myogenesis 40. Our data also proved that *HEY1* is a key factor involved in the tumorigenicity of osteosarcoma cells. We anticipate that this result will promote preclinical studies on this malignant tumor.

In summary, amino acid- and energy metabolism-associated genes are important to the tumorigenicity of osteosarcoma cells. Limited cell cycle progression and high level of amino acids and energy metabolism as well as aggrephagy may help osteosarcoma cells overcome the temporary nutrition deficiency and survive *in vivo*. Furthermore, *HEY1* is a key factor in the tumorigenicity of osteosarcoma cells. These results provide not only a new perspective to understand the mechanism of tumorigenicity but also another strategy for preclinical studies and targeted therapy of this orphan disease.

## Supplementary Material

Supplementary figures and table legend.

Supplementary table.
