# Supplementary material for: Identification of tumorigenicity-associated genes in osteosarcoma cell lines based on bioinformatic analysis and experimental validation
Source: J Cancer. 2020 Mar 26;11(12):3623–33. doi: 10.7150/jca.37393 (PMC7150450; doi:10.7150/jca.37393)
Supplement: Supplementary file 1 — Supplementary figures and table legend. [file jcav11p3623s1.pdf]

Supplementary Material

Table S1. Differentially expressed genes.

Figure S1. PPI analysis of lowly expressed genes in highly tumorigenic osteosarcoma cell lines.

Figure S2. PPI analysis of highly expressed genes in highly tumorigenic osteosarcoma cell lines.

Figure S1

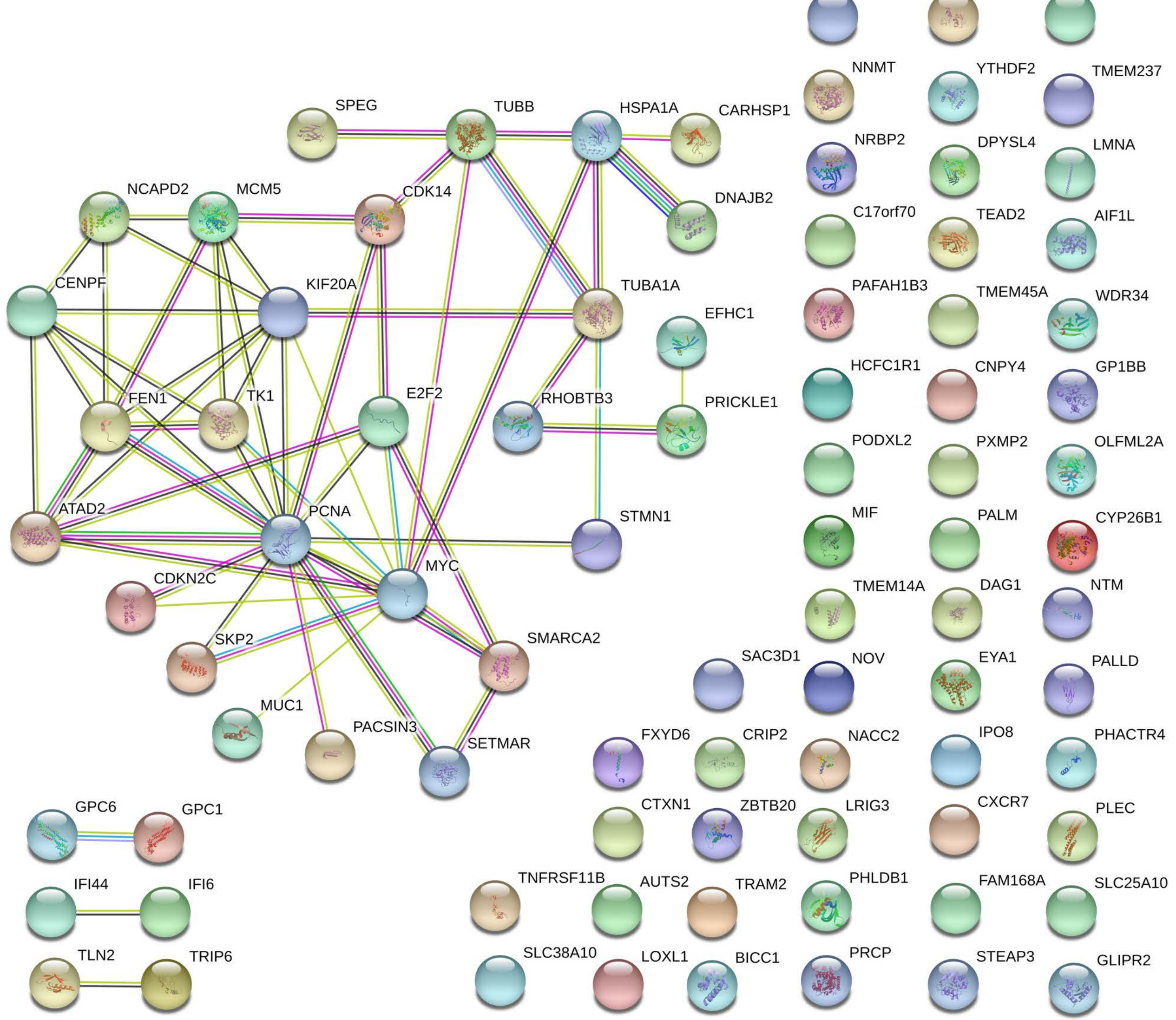

SPEG
TUBB
HSPA1A
CARHSP1
NCAPD2
MCM5
CDK14
DNAJB2
CENPF
KIF20A
TUBA1A
EFHC1
FEN1
TK1
E2F2
RHOBTB3
PRICKLE1
ATAD2
PCNA
STMN1
CDKN2C
MYC
SKP2
SMARCA2
MUC1
PACSIN3
SETMAR
GPC6
GPC1
IFI44
IFI6
TLN2
TRIP6
FAM198B
ZHX1
FAM111A
NNMT
YTHDF2
TMEM237
NRBP2
DPYSL4
LMNA
C17orf70
TEAD2
AIF1L
PAFAH1B3
TMEM45A
WDR34
HCFC1R1
CNPY4
GP1BB
PODXL2
PXMP2
OLFML2A
MIF
PALM
CYP26B1
TMEM14A
DAG1
NTM
SAC3D1
NOV
EYA1
PALLD
FXYD6
CRIP2
NACC2
IPO8
PHACTR4
CTXN1
ZBTB20
LRIG3
CXCR7
PLEC
TNFRSF11B
AUTS2
TRAM2
PHLDB1
FAM168A
SLC25A10
SLC38A10
LOXL1
BICC1
PRCP
STEAP3
GLIPR2

Figure S2

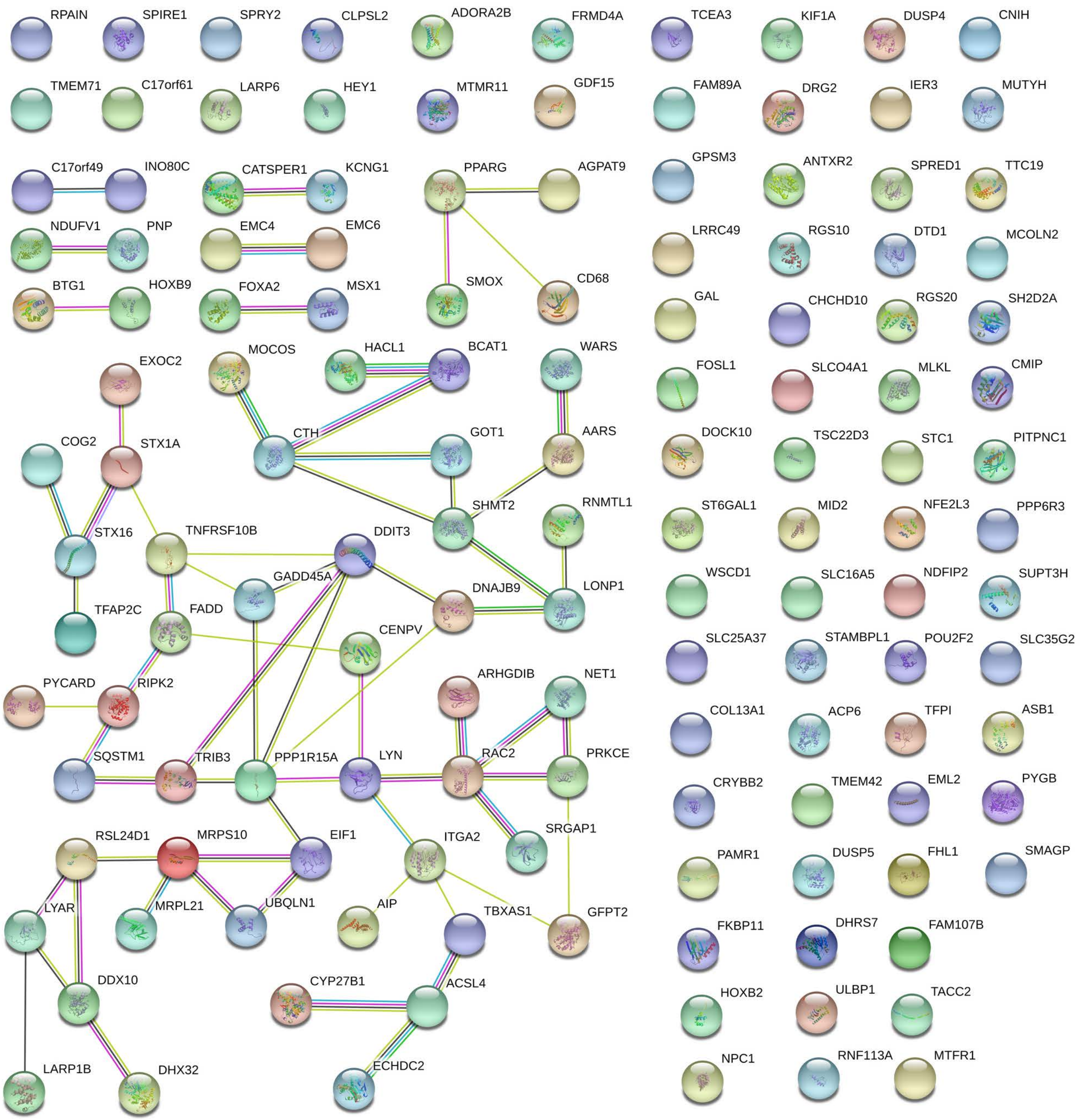

RPAIN
SPIRE1
SPRY2
CLPSL2
ADORA2B
FRMD4A
TCEA3
KIF1A
DUSP4
CNIH
TMEM71
C17orf61
LARP6
HEY1
MTMR11
GDF15
FAM89A
DRG2
IER3
MUTYH
C17orf49
INO80C
CATSPER1
KCNG1
PPARG
AGPAT9
GPSM3
ANTXR2
SPRED1
TTC19
NDUFV1
PNP
EMC4
EMC6
LRRC49
RGS10
DTD1
MCOLN2
BTG1
HOXB9
FOXA2
MSX1
SMOX
CD68
GAL
CHCHD10
RGS20
SH2D2A
EXOC2
MOCOS
HACL1
BCAT1
WARS
FOSL1
SLCO4A1
MLKL
CMIP
COG2
STX1A
CTH
GOT1
AARS
DOCK10
TSC22D3
STC1
PITPNC1
SHMT2
RNMTL1
ST6GAL1
MID2
NFE2L3
PPP6R3
STX16
TNFRSF10B
DDIT3
GADD45A
DNAJB9
LONP1
WSCD1
SLC16A5
NDFIP2
SUPT3H
TFAP2C
FADD
CENPV
SLC25A37
STAMBPL1
POU2F2
SLC35G2
ARHGDIB
NET1
PYCARD
RIPK2
COL13A1
ACP6
TFPI
ASB1
SQSTM1
TRIB3
PPP1R15A
LYN
RAC2
PRKCE
CRYBB2
TMEM42
EML2
PYGB
RSL24D1
MRPS10
EIF1
ITGA2
SRGAP1
PAMR1
DUSP5
FHL1
SMAGP
LYAR
MRPL21
UBQLN1
AIP
TBXAS1
GFPT2
FKBP11
DHRS7
FAM107B
DDX10
CYP27B1
ACSL4
HOXB2
ULBP1
TACC2
LARP1B
DHX32
ECHDC2
NPC1
RNF113A
MTFR1
